# A Reversal of the Association between Education Level and Obesity Risk during Ageing: A Gender-Specific Longitudinal Study in South Korea

**DOI:** 10.3390/ijerph17186755

**Published:** 2020-09-16

**Authors:** Woojin Chung, Roeul Kim

**Affiliations:** 1 Department of Health Policy and Management, Graduate School of Public Health, Yonsei University, Seoul 03722, Korea; wchung@yuhs.ac; 2 Institute of Health Services Research, Yonsei University, Seoul 03722, Korea; 3 Labor Welfare Research Institute, Korea Workers’ Compensation and Welfare Service, Seoul 07254, Korea

**Keywords:** obesity, education, ageing, gender, association, longitudinal study, women, South Korea

## Abstract

This study aimed to examine and quantify obesity risk across different education levels during ageing using the dataset of a nationally representative longitudinal survey. A total of 45,391 observations of 9991 individuals aged ≥45 years were included in this study. Obesity was defined as a body mass index of ≥25, according to a guideline for Asians by the World Health Organization, and education level was grouped into three categories. Socio-demographics, lifestyles, and health conditions were used as covariates. Adjusted odds ratios and predicted probabilities of obesity were computed and adjusted for a complex survey design. With respect to gender, education level and age were significantly associated with obesity risk, and the association was stronger in women than in men. Furthermore, education level was negatively associated with obesity risk in the middle age in each gender. However, the association became positive in the old age, specifically among highly educated women. Therefore, policy efforts to reduce obesity risk and the resulting education gradients should be established based on studies considering their old age. Further longitudinal studies are required to examine whether these findings are valid in other socio-cultural or economic settings.

## 1. Introduction

Obesity has been considered as one of the major causes of the onset and severity of chronic diseases [1], and if the ageing or aged population of a country had a high prevalence of obesity, it would be confronted with a heavy socioeconomic burden of an increasing number of chronic diseases [2] and a subsequent decrease in quality of life [3,4,5]. Personal characteristics such as demographic factors, socioeconomic status, lifestyle, and health conditions can determine eating and physical behaviours that lead to a greater risk of being overweight or obese. These obesity risk factors include low levels of education [6], blue-collar occupation [7], residing in urban areas [7], smoking [8], alcohol intake [9], routine physical exercise [10], psychosocial stress [11], and chronic diseases [12].

Indeed, regarding the factors associated with obesity risk, numerous studies have focussed on education level and investigated this association because of the possibility that a person’s level of formal education being established at early adulthood influences most aspects of their remaining lifetime. Despite some inconsistencies regarding the association, most studies in developed countries reported a so-called “inverse association between education level and obesity risk,” stating that education is negatively associated with obesity risk in both men and women, being more consistent in women than in men [13,14,15]. For example, a study carried out in Spain reported that obesity rate was highest among people with only elementary education for both men and women [16]. Another study in Luxembourg showed that, compared to women with a high level of education, women with primary or secondary education were twice as likely to be obese, thereby suggesting that a low level of education was a risk factor for obesity, particularly among women [17]. In addition, similar results were found in many developed countries such as France [18] and the USA [19]. Similar to studies conducted in the United States and European countries, a study performed in Japan found an inverse association between education level and being overweight among adolescents [20]. Another study performed in China showed that a significant inverse association between education level and obesity only existed in females; in males, income, rather than education level, was positively related to obesity [21].

Meanwhile, regarding the association between obesity and age, obesity is known to be highly prevalent in the middle-aged population [22,23]. For example, a study in the USA showed that obesity rate peaks in the middle age; it was found to be 8.5% among those aged 20–29 years, 21.4% among those aged 50–59 years, and 15.6% among those aged 70–79 years [22].

Examining the results of previous studies on obesity risk, we found that those studies used cross-sectional datasets rather than longitudinal datasets and failed to detect complex associations between education level and obesity risk during ageing. More precisely, although obesity risk and factors related to it can change during ageing, previous studies have failed to answer the question on whether the inverse associations between education level and obesity risk remain valid during ageing. Moreover, they have seldom investigated how the likelihood of obesity changes during ageing for each education level and whether this inverse association is the same by gender. From an academic viewpoint, it is important to examine the association between education level and obesity risk during ageing by gender to aid researchers in developing and testing new theories. This would also help policy-makers to design and implement effective policies to reduce obesity risk across the lifespan of both men and women of different education levels.

In this study, therefore, we aim to address a substantial gap in the literature and examine the association between education level and obesity risk during ageing in middle-aged and older adults with respect to gender. To achieve this, we used six waves of data from a Korean national longitudinal survey and conducted multivariable, mixed model analyses. In addition, we conducted a growth curve model analysis by estimating the predicted probability of obesity in an individual (man/woman) during ageing and compared them across education levels. Our interest in South Korea (hereafter, Korea) stems from the following reasons: (1) Korea is the 12th largest developed economy in the world [24]; (2) there has been a large difference in education level between genders, specifically among the older generation in Korea; the enrolment ratio of higher education in males relative to females tripled in the 1960s and 1970s, and became almost double in 1991 [25]; and (3) its population is rapidly ageing due to both its very effective, universal, public health insurance system and its remarkably low fertility rate; therefore, Korea is expected to become the world’s most aged society by 2067 [26].

## 2. Materials and Methods

### 2.1. Data Source and Study Sample

Our data source is the first six waves of the Korean Longitudinal Study of Ageing (KLoSA) survey, a nationally representative non-institutionalised, civilian population survey supervised by the Korean Ministry of Employment and Labour, which was conducted biennially from 2006 to 2016. The KLoSA survey used a stratified, multi-stage, clustered probability sampling design and collected data on South Koreans aged 45 years and over [27]. Following the ethical principles of the Declaration of Helsinki, informed consent forms were obtained from all participants. The KLoSA survey included information regarding participants’ sociodemographic, lifestyle, health-, and labour-related characteristics. In the first wave of the survey in 2006, 10,254 individuals completed a personal interview.

Our analysis was restricted to individuals who were surveyed during the first wave in 2006 to keep the same individuals in the later surveys. Of 47,995 observations, the following cases were excluded: non-contact, refusal or death (1474 observations); and non-report of the body mass index (1130 observations). The final study sample comprised 45,391 observations of 9991 participants, with an average of 4.46 observations per participant (standard deviation = 1.99; range = 1–6). We used the KLoSA datasets, which are publicly available from the KLoSA website [28], where detailed information about the survey design, procedure, and characteristics can be obtained. The Yonsei University Health System Institutional Review Board approved this study (Y-2019–0178).

### 2.2. Measurements

The body mass index (BMI) of each participant was calculated using data from self-reported height and weight values. According to the recommendation on Asia-Pacific criteria of obesity status given by the World Health Organization of the Western Pacific Region, we defined obesity as when an individual has a BMI of at least 25 kg/m^2^ [29].

The variables of interest were age, gender, and education level. For descriptive and univariable analysis, we categorised age into five groups (45–54, 55–64, 65–74, 75–84, and 85 years and above), but for multivariable analysis, we used a centred age (age minus its mean value) and its squared value to reduce a potential multicollinearity [30] and conduct a growth curve model analysis. Education level was grouped into three categories according to the highest level of formal education completed: (1) elementary school or less, (2) middle school or high school, and (3) college or higher.

As for covariates, we incorporated six sociodemographic characteristics: marital status (married and non-married, where non-married included never-married, separated, widowed, or divorced), religion (yes and no), residential area (urban and rural), occupation (no job, blue-collar job, and white-collar job), household income (lower half, higher half, and unreported), and housing tenure (house owner and house renter). We adjusted each wave’s household income for household size using the square root’s equivalence scale [31] and divided it into three groups: two groups were divided by their median values, and then the third group of participants did not report their household income; nevertheless, we retained the third group to avoid losing their other valuable information. As additional covariates, we included five characteristics about lifestyle and health conditions: smoking (smoking and non-smoking), alcohol intake (alcohol intake and non-alcohol intake), routine physical exercise activity (active and inactive), chronic disease (yes and no), and depressive symptoms (yes and no). We defined routine physical exercise activity based on self-reported answers to assess the participants’ engagement in any physical exercise at least once a week. We determined chronic disease according to self-reported answers to examine whether participants were diagnosed with chronic diseases (e.g., hypertension, diabetes, stroke, angina, myocardial infarction, chronic pulmonary diseases, and any type of cancer) by a physician. Depressive symptoms were defined as a score of four or more according to self-reported answers to survey questions on the 10-item short form of the Centre for Epidemiologic Studies Depression Scale (CES-D10) [32,33].

### 2.3. Analytic Procedures

Our analysis was based on three statistical models. Model 1 was a cross-sectional model that considered only participants at baseline (Wave 1). Model 2 was a longitudinal model with no covariates for all observations of all considered waves (1–6). As this is a longitudinal dataset, it is likely for observations to be temporally correlated within the same participant; hence, we employed a mixed logistic regression model with two levels: level 1 with observations and level 2 with participants. Furthermore, to avoid a potential bias in parameter estimates for the two-level mixed logistic regression models using small samples, we scaled the conditional weights at level 1 of the data hierarchy to normalise these conditional weights to sum up to within-level sample sizes [34,35]. Model 3 is another longitudinal model, but unlike Model 2, we included the covariates for all observations in all the considered waves in Model 3 and conducted the two-level mixed logistic regression model analyses.

After establishing these three statistical models, we then considered stratifying all analyses by gender. To do this, we tested the following null hypotheses: (1) if the obesity risk is the same between genders; (2) if the association between age and obesity risk is the same between genders; and (3) if the association between education level and obesity risk is the same between genders. We tested these hypotheses using a logistic model with main effect and interaction effect terms for participants in Models 1 to 3, employing the Chi-square and Wald tests. The results were as follows: the first hypothesis was rejected in Models 2 and 3 (*p* < 0.001) but not in Model 1 (*p* = 0.336); next, we rejected the second hypothesis in Models 1 to 3 (*p* < 0.001, *p* = 0.011, and *p* = 0.006 in Models 1–3, respectively); and finally rejected the third hypothesis in Models 1 to 3 (*p* < 0.001). In summary, because the tests of eight out of nine hypotheses in Models 1 to 3 (which assumed the null hypotheses of equality between genders) ended up being rejected, we decided to stratify all analyses by gender.

Furthermore, for multivariable analysis in Model 3, we took three steps to achieve well-fitting and parsimonious model specification. First, we continually re-categorised each of the variables and defined their reference categories differently, so that throughout all models, the values for the variance inflation factor was <2.21, exhibiting no considerable multicollinearity. Second, we used Pseudo Akaike’s information criterion as a measure of the goodness-fit of the mixed model and chose a random intercept model along with an unstructured diagonal covariance. The null model showed a high degree of intraclass correlation (0.917 and standard error 0.012 for men; 0.915 and standard error 0.011 for women), suggesting that, due to each model having a considerable degree of correlations between observations in an individual, it seemed appropriate to use the mixed model framework, just as our study did. Third, to have a parsimonious and compact model, this study included significant interaction effect terms between age and education level of all the variables of interest (age, age-squared, and education level).

We worried that due to the interaction effect terms between age and education level, it may be difficult to understand how a participant’s obesity risk with a particular education level, changes during ageing. To overcome this difficulty, using the results of the longitudinal model with all-studied covariates for each gender (Model 3), we estimated an individual’s predicted probability of being obese among those aged 45 to 90 years for each education level and obtained its 95% confidence intervals (CIs) through the delta method [36], thereby exhibiting growth curves of the predicted probability of obesity for each level of education during ageing. In summary, each predicted probability of obesity could be interpreted as a predicted value of the probability of obesity that a participant with a particular education level would have at a particular age, with all the other characteristics of the participants staying the same. The significant difference in the predicted probability of obesity between a certain level of education and another level of education at a specific age was evaluated by the Wald test.

For all estimation processes, we considered all characteristics as time-dependent (i.e., with the potential to change with time) and estimated the odds ratios (ORs) and 95% CIs, and we considered *p*-values < 0.05 (two-tailed) statistically significant. Statistical analyses were performed using SAS 9.4 software (SAS Institute, Cary, NC, USA) and STATA 15 software (StataCorp, College Station, TX, USA).

## 3. Results

Table 1 shows the characteristics of the participants at baseline (Wave 1) by gender. On average, men’s BMI is similar to that of women (23.1 vs. 23.2), and the mean age of women is higher than that of men (61.7 vs. 61.0 years). We found a higher proportion of women than men in the following characteristics categories: aged 75–84 years, aged 85 years and above, non-married, having a religion, residing in an urban area, attaining an education level of elementary school or less, having no job, belonging to the lower half group of household income, belonging to the group who did not report household income, house renter, non-smoking, non-alcohol-intake, inactive routine physical exercise, obese, having a chronic disease, and having a depressive symptom (Supplementary Table S1 exhibits sample characteristics at waves 2 to 6 by gender; Supplementary Table S2 demonstrates the number of participants and percentage experiencing the obesity status between waves by gender).

In Table 2, we present the prevalence rate of obesity across age categories and education levels at baseline by gender, obtained from Model 1 (the cross-sectional model), as well as the distribution of observations across age categories and education levels by each wave.

Overall, the prevalence rate of obesity was slightly higher in women (23.9%, 95% CI: 22.7% to 25.1%) than in men (22.9%, 95% CI: 21.5% to 24.4%), with no significant difference between genders (Rao-Scott Chi-square test, *p* = 0.336). However, the prevalence rate of obesity differed across age categories (Rao-Scott Chi-square test, *p* < 0.001) and had a negative, linear trend during ageing (Wald test, *p* < 0.001 in men and *p* = 0.016 in women) for each gender. The prevalence rate decreased rapidly from participants aged 45–54 years to those aged 85 years and above for each gender; from 25.1% (95% CI: 22.8 to 27.4%) to 12.9% (95% CI: 6.4 to 24.3%), respectively in men and from 22.5% (95% CI: 20.5 to 24.5%) to 4.5% (95% CI: 2.1 to 9.5%), respectively in women. Therefore, these results from Model 1 suggest that for each gender, obesity risk varies during ageing.

Regarding education, the prevalence rate of obesity varied across education levels (Rao-Scott Chi-square test, *p* = 0.003 in men and *p* < 0.001 in women). However, despite the significant linear trend from the lowest to the highest education level for each gender (*p* < 0.001), men showed a positive, linear trend, while women showed a negative, linear trend. Change in the prevalence rate from participants with an education level of elementary school or less to those with an education level of college or higher was from 18.9% (95% CI: 16.7 to 21.4%) to 26.2% (95% CI: 22.8 to 29.8%), respectively in men but from 26.2% (95% CI: 24.6 to 27.8%) to 10.1% (95% CI: 7.0 to 14.4%), respectively in women. Therefore, for Model 1, these results suggest that for each gender, the risk of obesity differs across education levels.

Table 3 shows the associations of age and education with obesity, obtained from the two longitudinal models. Model 2 has no covariates, and Model 3 is the one with all-studied covariates. The results from Model 2 in men and women are displayed in the first and second columns of Table 3, respectively. In the main effects terms, obesity risk was significantly associated with age for each gender (Wald test, *p* < 0.001 in men; *p* = 0.005 in women), showing a decrease with age; however, age-squared was significantly associated with obesity risk only in women (Wald test, *p* = 0.084 in men; *p* < 0.001 in women). For education level, obesity risk, being significantly associated with it for each gender (Wald test, *p* < 0.001), increased with education level in men, but decreased in women. Regarding interaction effect terms, we found that age and education level were interactively associated with the risk of obesity, where the association was stronger in women than in men (Wald test, *p* < 0.05). The results that we obtained after testing the significance of all the main effect and interaction effect terms relating to age, jointly for each gender suggest that in Model 2, obesity risk varies during ageing for each gender (Wald test, *p* < 0.001). The results of applying this method to education level in place of age suggest that in Model 2, obesity risk differs across education levels for each gender (Wald test, *p* < 0.001).

We present the results from Model 3 in men and women in the third and fourth columns of Table 3, respectively. In the main effects terms, the association of obesity risk with age was significant for each gender (Wald test, *p* < 0.001), where obesity risk decreased with age; however, the association of obesity risk with age-squared was significant only in women (Wald test, *p* = 0.176 in men; *p* < 0.001 in women). Additionally, obesity risk was significantly associated with education level only in women (Wald test, *p* = 0.228 in men; *p* < 0.001 in women), indicating that obesity risk decreased with education level among women. Interaction effect terms between age and education level were significant, being more strongly in women than in men, which is similar to the results of Model 2. The results obtained after testing the significance of all terms relating to age, jointly for each gender suggest that in Model 3, obesity risk varies during ageing for each gender (Wald test, *p* < 0.001). Additionally, the results regarding education level suggest that in Model 3, obesity risk differs across education levels for each gender (Wald test, *p* = 0.026 in men; *p* < 0.001 in women). For covariates, the following were significantly associated with obesity risk: residential area, smoking, alcohol intake, routine physical exercise, depressive symptoms, and chronic disease in men; and residential area, occupation, alcohol intake, and chronic disease in women.

**Figure 1:**
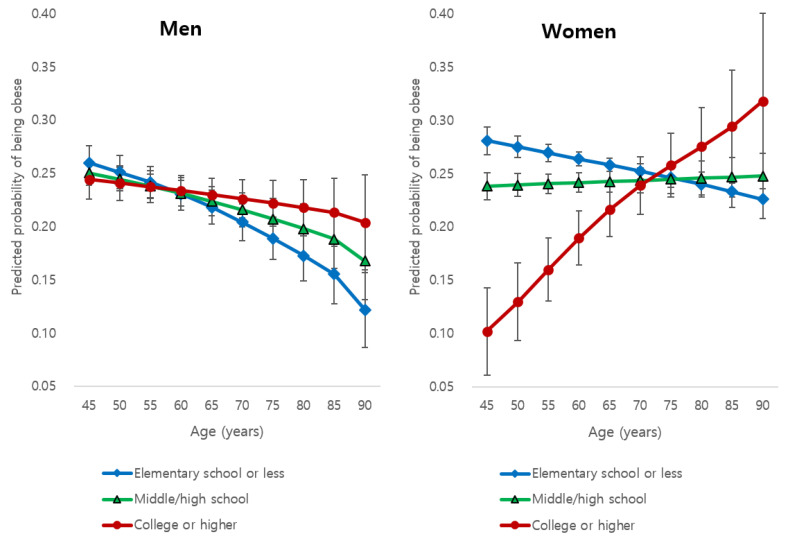
Gender-specific changes in the predicted probability of obesity during ageing for each education level and its 95% confidence interval.

Figure 1 shows gender-specific growth curves of the predicted probability of obesity for each education level and their 95% CIs during ageing (from age 45 to 90 years), which were estimated based on the results from Model 3.

In men, it is noteworthy that during ageing, the predicted probabilities of obesity tend to decrease more rapidly in the lower level of education category than in the higher level of education category. Precisely, at the age of 45 years, the predicted probabilities of obesity in the elementary school or less category (0.26; 95% CI 0.24 to 0.28) and the probability in the college or higher category (0.24; 95% CI 0.23 to 0.26) were very similar (Wald test for their difference, *p*-value = 0.310). However, at the age of 90 years, the probability was much higher in the college or higher category (0.21; 95% CI 0.17 to 0.25) than in the elementary school or less category (0.14; 95% CI 0.11 to 0.17)(Wald test for their difference, *p*-value = 0.030). This may suggest that in men, the association between education level and obesity risk changes from negative to positive during ageing.

Furthermore, in the case of women, during ageing, the predicted probabilities of obesity decreased in the elementary school or less category, whereas the probability increased in the other two categories with a higher level of education (slowly in the middle or high school category but rapidly in the college or higher category). At the age of 45 years, the predicted probabilities of obesity were significantly different between the elementary school or less category and the college or higher category (Wald test for their difference, *p*-value < 0.001), being almost three times higher in the former (0.28; 95% CI 0.27 to 0.29) than in the latter category (0.10; 95% CI 0.06 to 0.14); however, at the age of 90 years, the predicted probabilities were higher in the college or higher category (0.32; 95% CI 0.24 to 0.40) compared to the elementary school or less category (0.23; 95% CI 0.21 to 0.24), showing a significant difference (Wald test for their difference, *p*-value = 0.034). This suggests that in women, more clearly than in men, the association between education level and obesity risk changes from negative to positive during ageing.

## 4. Discussion

In this study, we investigated the gender-specific association between education level and obesity risk during ageing in middle-aged and older adults using six waves of data from a Korean national longitudinal survey. Using one cross-sectional model (Model 1) and two longitudinal models (Models 2 and 3), we found that obesity risk varied both with education levels and during ageing from middle age to old age, and the association between education level and obesity risk during ageing differed between genders. More specifically, we noted a reversal of the inverse association between education level and obesity risk during ageing, which was much stronger in women than in men. This suggests that in middle age, the risk of obesity is higher in the lower education group than in the higher education group—the inverse association between education level and obesity risk; however, in old age, the risk of obesity is higher in the higher education group than in the lower education group—a reversal of the inverse association between education level and obesity risk.

The inverse association between education level and obesity risk, which this study exhibited in middle age, is consistent with the results of numerous previous studies carried out in developed countries, which used cross-sectional datasets [13,14,16,17,18,19,37,38,39,40,41,42,43,44,45,46,47,48,49]. However, there are studies (either developed or developing countries), showing no inverse associations between education level and obesity risk for both genders: for the USA, positive in men but negative in women [50], insignificant in men but negative in women [51], or positive in men but insignificant in women [52]; for Italy, insignificant in men but negative in women [53]; for The Netherlands, insignificant in both men and women [54]; for Finland, insignificant in both men and women [55]; for Finland, positive in both men and women [56]; in India, positive in both men and women; [57] for Iran, positive in men but negative in women [58]; for Peru, positive in men but insignificant in women [59].

It is unfortunate that due to the limited number of studies that have used longitudinal datasets, we could not compare the major results of this study, which is a reversal of the inverse association between education level and obesity risk may occur during ageing, with those of other studies. However, some studies, despite their use of cross-sectional datasets, found that compared to less educated older people, highly educated older people were more likely to be obese, especially in developing countries. A study of people aged 60 years and over in Brazil reported that obesity was more common among people with higher educational levels [60]. According to a study in India comprising older adults obtained from the first wave of the WHO’s study in global AGEing and adult health (SAGE), overweight and obesity were more prevalent in wealthier and educated older adults [61]. A study that used the first wave of SAGE showed that educated older adults were more likely to be in a higher BMI category in China, Ghana, India, Mexico, Russia, and South Africa [62]. Using the data from the World Health Organization World Health Survey and analysing the older adult population in 70 low- and middle-income countries, a study found that older adults with higher education levels were more likely to be overweight or obese in the least urbanised countries, while the opposite was the case in most urbanised countries [63]. Meanwhile, using the National Health and Nutrition Examination Surveys analysing adults aged 20–66 years, a study in the USA found that the inverse association between education level and obesity risk has weakened over the past three decades [64].

In this study, using a longitudinal dataset, we found that obesity risk decreased during ageing from middle age to old age for each gender, which is in line with previous studies [22,65,66] except for the case of highly educated women. A plausible mechanism for the decrease in obesity risk during ageing seems to be related to unintentional weight loss among old adults, with an annual incidence of 13% [67]; at very old ages, individuals tend to lose muscle mass due to inactivity, reduction in energy intake, or illness [65,68]. In addition, one of our findings—the rate of decrease in obesity risk during ageing was slower in women than in men—seems similar to the results of previous studies [69,70,71]. A cross-sectional study in Italy reported that BMI was significantly higher in women than in men among people aged 65-84 years [71]. This is in part reported due to gender differences in adipose tissue storage and metabolism [72], because a decrease in estrogen after menopause may reduce metabolic activity, leading to more fat accumulation in elderly women than in elderly men [71,72]. However, the decrease in obesity risk during ageing has not been clearly observed in previous studies using cross-sectional datasets. For example, a study in Germany showed that the prevalence of overweight in people aged 80 years and over was lower than that in younger adults [23]. By contrast, a systematic review of 72 papers (1999–2019) showed that the prevalence of obesity increases among those aged 69 years in both men and women and then tends to decrease [73]. Future research should use longitudinal datasets to examine the association of obesity risk during ageing more rigorously.

This study found that the inverse association between education level and obesity risk turned out to be reversed during ageing from middle age to old age, being more strongly in women than in men. Although some studies indicated that obesity differentials by education level decline with age [64,68], no study demonstrated the reversal of the inverse association between education level and obesity risk, as we did in this study. We may establish a hypothesis to explore the reason for the results, stating that efforts to control body weight and maintain thinness to reach a high place of social position would differ between highly educated people and less educated people. According to social position theory [74,75], a place of social position can be reached through both “ascribed status” and “achieved status,” where “ascribed status”—for example, gender, parents’ wealth, and country of birth—is given to an individual before birth or at birth; however, “achieved status” can be obtained after birth through the individual’s efforts. In early adulthood and middle age, everything else being equal, highly educated people, who invested more time and resources to their education and sought their due returns, tend to desire more ardently to reach a higher place of social position than less educated people. Such ardent desires may result in them putting in extra efforts to achieve physical thinness; whereas in middle age, the highly educated people seem less likely to be obese than the less educated ones. In their old age, however, after they obtain a higher place of social position, they no longer feel the need to make great efforts as they did previously; hence, they relent on their efforts and begin to gain bodyweight, causing them to become obese. Indeed, there has been much evidence of social pressures for attaining thinness, especially for women, even in developed countries [76,77,78].

The notable finding in this study revealed that for highly educated women, their obesity risk tends to increase during ageing from middle age to old age. A tip for the answer to this might be for us to apply the social position theory mentioned previously to Korea, which has long been a “son-preferring” society with a male-dominated labour market. Indeed, in Korea, whose people have long preferred sons over daughters [79,80,81], most girls start from a lower place of “ascribed status” at birth than boys. Opportunity to achieve a high level of education from childhood to early adulthood is likely to be granted to boys rather than girls by their son-preferring parents and the society to which they belong [25]; therefore, given the scarcity of family resources, girls need to make greater efforts than boys to satisfy their parents and society to catch the opportunity. After completing a certain level of education, this is similar in the labour market. Korea has been long noted for its male-dominated labour market [82,83]. Because of the familial and societal pressures toward women to be primary homemakers or caregivers, women tend to quit their work in the labour market once they get married or have children [82,84]. According to the dataset of the Organization for Economic Cooperation and Development, in Korea, the female employment rate is around 56.1%, which is far below the male employment rate of 75.9% [82]. Usually, in such a male-dominated labour market, women’s thinness or beauty is known to be valued as a symbolic asset, which translates into tangible social resources [85,86]. Thus, local culture and norms put great pressure on women to lose weight [87,88,89]. Therefore, in Korea, compared to their male counterparts, highly educated women put more efforts into controlling their body weight to fit themselves in such a labour market, so that they may get a job, survive, or get promoted. In Korea, many cases have reported that highly educated women suffer from sexual harassment or rape by their bosses in their workplaces, even in the public sector [90,91,92]. In addition, even in marriage, highly educated women tend to be very concerned about their body weight in consideration of their potential marriage partners and parents-in-law because in a higher class in society, obese women tend to be more judged than in a lower class society [86,93]. As a result, given that everything else is equal, women may make greater efforts than males do to reach a high place of social position until their middle age. It is likely that approaching old age, highly educated women, whether they reach their desired place of social position or not, try to be freed from the deep-rooted, mental, and physical fetters of social pressures toward thinness, and end up enjoying their freedom and gaining weight in their old age.

Indeed, numerous studies provide evidence of young women’s great efforts to avoid overweight and obesity in Korea. According to a cross-country study of young, educated women in 22 countries, the age-adjusted prevalence of seeing oneself as overweight was the highest in Korean women (77%) [87]. Another study states that women’s social value is closely related to their thinness in Korea [47]. One recent study, using datasets from the Korea National Health and Nutrition Examination Survey and exploring the factors contributing to educational differences in obesity in Korean women aged 25 years or over through an extended Oaxaca-Blinder decomposition method, showed that the difference in the level of perceived stress between the highly educated women group and the less educated women group was the most important contributor to the difference in obesity risk between the two groups of women [94]. According to these, it seems definite that further research is required to test our hypothesis using both quantitative and qualitative methods in other settings, in particular, for either son-preferred or male-dominated societies such as China, India, and Japan.

From a policy perspective, although discretion must be exercised in drawing policy suggestions, this study suggests that increased formal education may reduce obesity risk in young or middle-aged adults but increase in old age, especially for highly educated women. This could get many policy-makers to discuss the issue of whether or not a formal education needs to be enhanced to reduce the prevalence of obesity, thereby helping them to be healthier and live longer [95,96,97,98].

Meanwhile, obese elderly individuals reported more doctor visits and perceived their health to be worse than normal-weight elderly individuals [60]. Obese elderly individuals are especially vulnerable to metabolic derangements [99] and face functional limitations that lead to a cycle of inactivity, further weight gain, and functional deterioration [100]. Therefore, evidence-based weight-loss programmes should be tailored according to individual needs to provide a balanced diet, an appropriate level of calorie intake, and safe physical activity for obese elderly individuals [101]. However, health education and interventions are necessary for proper weight and body perception for highly educated young women with inappropriate weight-loss behaviours [102]. Many studies have shown that obesity misperception harms happiness and that body perception along with a normal and proper weight are important [102,103,104].

Additionally, regarding the associations of covariates with the risk of obesity in the present study, residing in urban areas, alcohol intake, and having chronic diseases were associated with the risk of obesity in both males and females. Occupation was associated with the risk of obesity only in females. Smoking, routine physical exercise, and depressive symptoms were associated with the risk of obesity only in males. However, no study has performed a multi-dimensional analysis of the associations between these variables and education level in relation to obesity, except one recent study that dealt with the associations between lifestyle behaviours and education level relation to obesity [105]. This study considered a total of six lifestyle behaviours—smoking status, the risk from drinking alcohol, physical exercise activity, daily sleep duration, daily energy intake, and level of stress—and reported that the modifying effects of education level on the associations between lifestyle behaviours and obesity depend on both sex and lifestyle behaviour [105]. Similar to this study, future studies need to elucidate how a person’s characteristics influence, either as a confounder or an effect modifier, the association between a person’s education level and risk of obesity.

### 4.1. Strengths

To the best of our knowledge, this is the first study to investigate the gender-specific association between education level and obesity risk during ageing in middle-aged and older adults using a nationally representative longitudinal dataset and time-varying covariates in the mixed model analysis. This study noted that despite an inverse association between education level and obesity risk in middle age, the association could be reversed in old age; in particular, for highly educated women, obesity risk may increase during ageing. As for the generalisability of our research findings, the method used can be applied to other socio-cultural settings, and the results can be tested because our research included a broad range of participants from a nationally representative longitudinal sample of the South Korean population aged 45 years and over through the KLoSA survey.

### 4.2. Limitations

This study has some limitations. First, obesity estimates from the KLoSA survey are derived from self-reported height and weight data, which may have led to measurement error. Second, there were 1474 missing observations due to non-contact, refusal, or death and 1130 missing observations due to non-reporting of the body mass index in the present study. However, because the percentage of these missing observations from the total observations was relatively low (5.7% of 47,995 observations), we decided to analyse the remaining 45,391 observations without performing an imputation method that might be subject to other types of statistical problems [106]. Future research needs to investigate whether this might result in biased estimates and a loss of power. Third, given the lack of related information, this study did not consider other potential covariates, such as quality of education, peer network, genetics, parity, parental obesity, dietary intake, and aerobic and muscle-strengthening physical activities. Fourth, for expository convenience, this study precluded the interactions between education level and other covariates. Some recent studies found that education plays a role as either a confounder or modifier in the associations between other socioeconomic status indicators (or health lifestyles) and obesity risk [105,107]. Fifth, because of the small number of participants with college or higher level of education in older people, particularly for women, the CIs of the predicted probability of obesity in participants with college or higher level of education tended to widen as they got older. In addition, because only 4% of women were college educated, we should interpret the results with cautions. Future studies need to use a larger study sample, including highly educated older women, to obtain more precise educational differences in obesity risk even at a very old age. Sixth, the scope of the study is limited by the cultural customs and ways of life of each country. Seventh, we could not consider the possibility of skewness in the study sample because of risk of ealier death in obese persons. Obese persons are more likely to develop cardiovascular disease and earlier death [108]. Obesity is also associated with the leading causes of death in the United States and worldwide, including diabetes, heart disease, stroke, and some types of cancer [109]. Future studies need to use a larger study sample, including highly educated older women, to obtain more precise educational differences in obesity risk even at a very old age. Sixth, the scope of the study is limited by the cultural customs and ways of life of each country. Finally, although it is beyond the scope of this study, it would be of great interest to incorporate characteristics such as race, ethnicity, or immigrant status into our analysis. However, the KLoSA survey does not include such information.

## 5. Conclusions

Presently, considering that there was no rigorous study of the association between education level and obesity risk during ageing, this is the first study to investigate the gender-specific association of education level with obesity status during ageing using a mixed model analysis of a nationally representative longitudinal dataset. The results from this study suggest that the association between education level and obesity risk may change from a negative sign to a positive sign during ageing in both men and women, and that policy efforts to reduce obesity risk and the resulting education gradients should be established based on studies considering their old age. Moreover, further research is required to examine whether the findings in this study remain valid in other settings in terms of either socio-cultural or economic development.

## Figures and Tables

**Table 1:** Sample characteristics by gender at baseline (Wave 1).

Characteristics	Men	Women
Body mass index: Mean (SD) ^a^	23.1 (2.6)	23.2 (3.0)
Age, years: Mean (SD) ^a^	61.0 (10.5)	61.7 (11.4)
45–54	32.9%	33.0%
55–64	28.7%	26.8%
65–74	26.9%	25.0%
75–84	10.2%	12.6%
85 and above	1.3%	2.6%
Non-married ^b^	8.2%	31.9%
Resides in rural area	22.6%	22.5%
Religion, yes	44.7%	63.7%
Education level		
Elementary school or less	31.2%	57.5%
Middle/high school	51.1%	37.5%
College or higher	17.7%	5.0%
Occupation		
No job	44.3%	76.2%
Blue collar job	39.9%	20.3%
White collar job	15.8%	3.5%
Household income ^c^		
Lower half	44.5%	47.5%
Higher half	48.6%	43.9%
Unreported	6.9%	8.6%
House renter	22.0%	24.2%
Smoking, yes	40.0%	3.1%
Alcohol intake, yes	63.1%	18.5%
Routine physical exercise, active	42.8%	35.6%
Obese ^d^, yes	21.5%	23.9%
Depressive symptom ^e^, yes	25.0%	35.9%
Chronic disease ^f^, yes	38.9%	39.4%
Number of observations	4399	5592

^a^ SD denotes standard deviation. ^b^ Nonmarried includes never married, separated, widowed, or divorced. ^c^ Household income was adjusted for household size for each wave. ^d^ Obese was defined as the body mass index of at least 25. ^e^ Depressive symptom was defined as a score of 4 or more on the 10-item short form of the Center for Epidemiologic Studies Depression Scale. ^f^ Chronic diseases include hypertension, diabetes, stroke, angina, myocardial infarction, chronic pulmonary diseases, and any type of cancer.

**Table 2:** Prevalence of obesity across all age groups and education levels by gender at baseline (Wave 1), and the distribution of observations across all age groups and education levels by each wave.

Characteristics	Prevalence (%)				Distribution (%)						
	Men		Women								
	Rate	(95% CI)	Rate	(95% CI)	Wave 1 (2006)	Wave 2 (2008)	Wave 3 (2010)	Wave 4 (2012)	Wave 5 (2014)	Wave 6 (2016)	Overall
Overall	22.9	(21.5–24.4)	23.9	(22.7–25.1)							
Chi-squared test, *p*-value	0.336										
Age, years											
45–54	25.1	(22.8–27.4)	22.5	(20.5–24.5)	32.9	26.4	20.0	13.6	7.0	0.0	18.6
55–64	24.9	(22.4–27.5)	28.9	(26.6–31.4)	27.7	28.0	30.1	31.8	33.0	31.2	30.0
65–74	17.5	(15.3–19.9)	24.9	(22.6–27.4)	25.8	28.7	30.0	30.9	31.6	32.6	29.5
75–84	13.4	(10.4–17.1)	17.6	(14.8–20.7)	11.6	13.8	16.1	19.6	22.9	27.6	17.7
85 and above	12.9	(6.4–24.3)	4.5	(2.1–9.5)	2.0	3.1	3.8	4.2	5.5	8.6	4.2
Chi-squared test, *p*-value		<0.001		<0.001						<0.001	
Linear trend test, *p*-value		<0.001		0.016							
Education level											
Elementary school or less	18.9	(16.7–21.4)	26.2	(24.6–27.8)	45.9	46.7	46.3	45.3	45.0	43.8	45.6
Middle/high school	23.5	(21.6–25.5)	23.2	(21.3–25.2)	43.5	43.4	43.8	44.6	44.9	47.3	44.4
College or higher	26.2	(22.8–29.8)	10.1	(7.0–14.4)	10.6	9.9	9.8	10.1	10.1	8.9	10.0
Chi-squared test, *p*-value		0.003		<0.001						<0.001	
Linear trend test, *p*-value		<0.001		<0.001							
Number of observations	4399		5592		9991	8502	7570	6865	6465	5998	45,391

**Table 3:** Longitudinal analyses of the associations of age and education with obesity risk by gender.

Characteristics	Men				Women				Men				Women			
	OR ^a^	(95% CI) ^b^		*p*	OR ^a^	(95% CI) ^b^		*p*	OR ^a^	(95% CI) ^b^		*p*	OR ^a^	(95% CI) ^b^		*p*
*Main effects*																
Age	0.95	(0.93–0.97)		<0.001	0.98	(0.97–0.99)		0.005	0.94	(0.92–0.96)		<0.001	0.96	(0.95–0.98)		<0.001
Age-squared	0.999	(0.998–1.000)		0.084	0.998	(0.997–0.999)		<0.001	0.999	(0.998–1.000)		0.175	0.998	(0.997–0.999)		<0.001
Educational level (Ref: Elementary school or less)																
Middle/high school	1.48	(1.17–1.86)		0.001	0.72	(0.58–0.89)		0.002	1.14	(0.90–1.45)		0.273	0.63	(0.51–0.78)		<0.001
College or higher	2.04	(1.51–2.77)		<0.001	0.35	(0.21–0.59)		<0.001	1.33	(0.96–1.85)		0.086	0.33	(0.19–0.56)		<0.001
*Interaction effects*																
Middle/high school*Age	1.03	(1.00–1.05)		0.055	1.04	(1.02–1.07)		<0.001	1.02	(1.00–1.05)		0.093	1.04	(1.02–1.07)		<0.001
College or higher*Age	1.04	(1.00–1.07)		0.032	1.16	(1.10–1.23)		<0.001	1.04	(1.01–1.08)		0.016	1.16	(1.10–1.22)		<0.001
*Covariates*																
Non-married ^c^ (Ref: Married)									0.91	(0.65–1.25)		0.550	0.90	(0.73–1.10)		0.294
Resides in rural area (Ref: Reside in urban area)									0.59	(0.46–0.76)		<0.001	0.50	(0.41–0.61)		<0.001
Religion (Ref: No)									1.19	(1.00–1.41)		0.050	1.03	(0.90–1.18)		0.672
Occupation (Ref: No job)																
Blue collar job									0.90	(0.72–1.13)		0.362	0.81	(0.68–0.98)		0.027
White collar job									1.21	(0.85–1.72)		0.285	0.69	(0.43–1.11)		0.123
Household income ^d^, higher half (Ref: Lower half and unreported)									1.06	(0.89–1.27)		0.493	1.00	(0.87–1.15)		0.975
House renter (Ref: Owner)									0.96	(0.76–1.23)		0.756	1.01	(0.83–1.22)		0.920
Smoking, yes (Ref: Not smoking)									0.68	(0.55–0.84)		<0.001	0.66	(0.40–1.08)		0.095
Alcohol intake, yes (Ref: Not alcohol intake)									1.46	(1.18–1.81)		<0.001	1.38	(1.10–1.74)		0.005
Active, routine physical exercise (Ref: Not active)									1.21	(1.03–1.43)		0.020	0.98	(0.85–1.13)		0.785
Depressive symptom ^e^, yes (Ref: No)									0.68	(0.59–0.80)		<0.001	0.98	(0.87–1.11)		0.764
Chronic disease ^f^, yes (Ref: None)									2.08	(1.68–2.58)		<0.001	2.67	(2.22–3.21)		<0.001
Number of observations	19,707				25,684				19,707				25,684			

Effect of a continuous variable, age, was centered around its mean and assessed as one unit offset from its centered mean. All values were estimated with a complex sampling design. All characteristics were considered to be time-dependent. ^a^ OR denotes odds ratio. ^b^ CI denotes confidence interval. ^c^ Nonmarried included never married, separated, widowed, or divorced. ^d^ Household income was adjusted for household size for each wave. ^e^ Depressive symptom was defined as a score of 4 or more on the 10-item short form of the Center for Epidemiologic Studies Depression Scale. ^f^ Chronic diseases include hypertension, diabetes, stroke, angina, myocardial infarction, chronic pulmonary diseases, and any type of cancer.

## Supplementary Materials

The following are available online at https://www.mdpi.com/1660-4601/17/18/6755/s1, Table S1: Sample characteristics at Waves 2 to 6 by gender. Table S2: Changes in the obesity status between waves by gender (the number of participants and percentage).
