# Supplementary material for: A Reversal of the Association between Education Level and Obesity Risk during Ageing: A Gender-Specific Longitudinal Study in South Korea
Source: Int J Environ Res Public Health. 2020 Sep 16;17(18):6755. doi: 10.3390/ijerph17186755 (PMC7559988; doi:10.3390/ijerph17186755)
Supplement: Supplementary file 1 [file ijerph-17-06755-s001.pdf]

Supplementary Table S1. Sample characteristics at waves 2 to 6 by gender.

Characteristics	Men					Women				
	Wave 2 (2008)	Wave 3 (2010)	Wave 4 (2012)	Wave 5 (2014)	Wave 6 (2016)	Wave 2 (2008)	Wave 3 (2010)	Wave 4 (2012)	Wave 5 (2014)	Wave 6 (2016)
Body mass index: Mean (SD) [a]	23.1 (2.6)	23.1 (2.6)	23.1 (2.6)	23.1 (2.6)	23.2 (2.5)	23.2 (2.9)	23.3 (2.9)	23.3 (2.9)	23.3 (2.9)	23.4 (2.9)
Age, years: Mean (SD) [a]	63.1 (10.3)	64.7 (10.0)	66.4 (9.6)	67.9 (9.4)	70.5 (9.1)	63.7 (11.2)	65.3 (10.9)	66.8 (10.4)	68.4 (10.1)	70.8 (9.8)
45-54	26.0%	19.2%	12.7%	6.5%	0.0%	26.7%	20.6%	14.2%	7.4%	0.0%
55-64	28.7%	29.8%	31.8%	32.6%	33.5%	26.6%	28.8%	31.1%	32.7%	31.7%
65-74	29.5%	31.2%	32.1%	33.2%	34.2%	28.1%	29.1%	29.9%	30.3%	31.4%
75-84	12.6%	15.1%	19.4%	22.5%	27.9%	14.8%	16.8%	19.8%	23.1%	27.4%
85 and above	2.1%	2.7%	3.3%	4.3%	7.3%	3.8%	4.7%	5.0%	6.5%	9.5%
Non-married [b]	8.6%	8.8%	9.1%	9.3%	9.5%	32.4%	33.2%	33.1%	34.5%	35.8%
Resides in rural area	24.5%	25.3%	25.9%	26.0%	26.0%	24.3%	25.5%	26.1%	26.8%	27.1%
Religion, yes	40.5%	38.9%	39.9%	33.3%	33.2%	58.8%	56.5%	59.0%	51.2%	48.9%
Education level										
Elementary school or less	31.8%	31.2%	30.4%	29.6%	28.2%	58.2%	57.9%	56.8%	56.6%	55.4%
Middle/high school	51.2%	52.0%	52.3%	52.9%	56.2%	37.4%	37.6%	38.7%	38.9%	40.7%
College or higher	17.0%	16.9%	17.4%	17.5%	15.6%	4.4%	4.5%	4.5%	4.5%	3.9%
Occupation										
No job	44.6%	43.8%	48.3%	51.3%	53.4%	71.8%	70.6%	72.9%	74.6%	75.9%
Blue collar job	41.6%	43.7%	40.4%	38.6%	37.8%	25.0%	26.0%	24.1%	22.9%	21.9%
White collar job	13.8%	12.5%	11.3%	10.1%	8.9%	3.2%	3.4%	3.0%	2.5%	2.2%
Household income [c]										
Lower half	46.6%	46.8%	46.3%	46.2%	48.4%	52.0%	52.0%	52.4%	52.1%	54.3%
Higher half	52.1%	52.7%	53.3%	53.1%	51.5%	46.0%	47.3%	47.0%	46.8%	45.4%
Unreported	1.4%	0.5%	0.4%	0.7%	0.1%	2.1%	0.7%	0.6%	1.2%	0.3%
House renter	17.9%	17.3%	16.7%	15.7%	15.1%	19.6%	19.0%	18.0%	17.1%	16.4%
Smoking, yes	37.6%	36.3%	33.8%	27.6%	22.0%	3.2%	2.8%	2.5%	1.9%	1.6%
Alcohol intake, yes	60.3%	58.0%	56.1%	55.1%	53.7%	18.4%	18.0%	17.6%	16.0%	15.4%
Routine physical exercise, active	39.5%	37.9%	41.3%	36.8%	39.4%	33.0%	30.5%	32.1%	29.2%	30.5%
Obese [d], yes	18.8%	19.8%	20.9%	20.2%	21.2%	23.8%	25.2%	25.2%	25.9%	26.6%
Depressive symptom [e], yes	40.4%	41.2%	40.4%	37.5%	34.6%	52.2%	49.8%	46.4%	44.6%	41.5%
Chronic disease [f], yes	44.9%	49.3%	53.5%	56.5%	59.3%	45.3%	51.2%	55.0%	58.7%	61.1%
Number of observations	3,711	3,281	2,983	2,777	2,556	4,791	4,289	3,882	3,688	3,442

[a] SD denotes standard deviation. [b] Nonmarried includes never married, separated, widowed, or divorced. [c] Household income was adjusted for household size for each wave. [d] Obese was defined as the body mass index of at least 25. [e] Depressive symptom was defined as a score of 4 or more on the 10-item short form of the Center for Epidemiologic Studies Depression Scale. [f] Chronic diseases include hypertension, diabetes, stroke, angina, myocardial infarction, chronic pulmonary diseases, and any type of cancer.

Supplementary Table S2. Changes in the obesity status between waves by gender (the number of participants and percentage).

Men

		Wave 2 Not obese	Wave 2 Obese	Total
**Wave 1**	Not obese	2,704 (93.9%)	177 (6.1%)	2,881 (100.0%)
	Obese	275 (34.6%)	520 (65.4%)	795 (100.0%)
	Total	2,979 (81.0%)	697 (19.0%)	3,676 (100.0%)

		Wave 3 Not obese	Wave 3 Obese	Total
**Wave 2**	Not obese	2,421 (91.8%)	216 (8.2%)	2,637 (100.0%)
	Obese	188 (30.3%)	432 (69.7%)	620 (100.0%)
	Total	2,609 (80.1%)	648 (19.9%)	3,257 (100.0%)

		Wave 4 Not obese	Wave 4 Obese	Total
**Wave 3**	Not obese	2,226 (94.7%)	125 (5.3%)	2,351 (100.0%)
	Obese	109 (18.2%)	490 (81.8%)	599 (100.0%)
	Total	2,335 (79.2%)	615 (20.9%)	2,950 (100.0%)

		Wave 5 Not obese	Wave 5 Obese	Total
**Wave 4**	Not obese	2,105 (97.1%)	64 (3.0%)	2,169 (100.0%)
	Obese	87 (15.0%)	495 (85.1%)	582 (100.0%)
	Total	2,192 (79.7%)	559 (20.3%)	2,751 (100.0%)

		Wave 6 Not obese	Wave 6 Obese	Total
**Wave 5**	Not obese	1,918 (94.9%)	104 (5.1%)	2,022 (100.0%)
	Obese	90 (17.1%)	435 (82.9%)	525 (100.0%)
	Total	2,008 (78.8%)	539 (21.2%)	2,547 (100.0%)

Women

		Wave 2 Not obese	Wave 2 Obese	Total
**Wave 1**	Not obese	3,225 (91.2%)	311 (8.8%)	3,536 (100.0%)
	Obese	337 (29.5%)	805 (70.5%)	1,142 (100.0%)
	Total	3,562 (76.1%)	1,116 (23.9%)	4,678 (100.0%)

		Wave 3 Not obese	Wave 3 Obese	Total
**Wave 2**	Not obese	2,869 (89.8%)	327 (10.2%)	3,196 (100.0%)
	Obese	285 (27.7%)	744 (72.3%)	1,029 (100.0%)
	Total	3,154 (74.7%)	1,071 (25.4%)	4,225 (100.0%)

		Wave 4 Not obese	Wave 4 Obese	Total
**Wave 3**	Not obese	2,679 (94.5%)	155 (5.5%)	2,834 (100.0%)
	Obese	173 (17.6%)	808 (82.4%)	981 (100.0%)
	Total	2,852 (74.8%)	963 (25.2%)	3,815 (100.0%)

		Wave 5 Not obese	Wave 5 Obese	Total
**Wave 4**	Not obese	2,578 (95.3%)	126 (4.7%)	2,704 (100.0%)
	Obese	107 (11.5%)	820 (88.5%)	927 (100.0%)
	Total	2,685 (74.0%)	946 (26.1%)	3,631 (100.0%)

		Wave 6 Not obese	Wave 6 Obese	Total
**Wave 5**	Not obese	2,367 (93.7%)	159 (6.3%)	2,526 (100.0%)
	Obese	149 (16.5%)	752 (83.5%)	901 (100.0%)
	Total	2,516 (73.4%)	911 (26.6%)	3,427 (100.0%)
